# Prophylactically Feeding Manganese to *Drosophila* Confers Sex-Specific Protection from Acute Ionizing Radiation Independent of MnSOD2 Levels

**DOI:** 10.3390/antiox14020134

**Published:** 2025-01-23

**Authors:** Robert P. Volpe, Aditya Sen, Ajay Sharma, Venkatesan Kathiresan, Brian M. Hoffman, Rachel T. Cox

**Affiliations:** 1 Department of Biochemistry and Molecular Biology, Uniformed Services University, Bethesda, MD 20814, USA; robert.volpe.ctr@usuhs.edu (R.P.V.); aditya.sen.ctr@usuhs.edu (A.S.); 2 Henry M. Jackson Foundation for the Advancement of Military Medicine, Bethesda, MD 20817, USA; 3 Department of Chemistry, Northwestern University, Evanston, IL 60208, USA; ajay-sharma@northwestern.edu (A.S.); venkatesan.kathiresan@northwestern.edu (V.K.); bmh@northwestern.edu (B.M.H.)

**Keywords:** ionizing radiation, radiation protection, manganese, electron paramagnetic resonance (EPR), electron-nuclear double resonance (ENDOR), SOD2, lifespan, *Drosophila*

## Abstract

Ionizing radiation is a health threat to many, including warfighters, radiological emergency responders, radiotherapy patients, and astronauts. Despite this, no FDA-approved prophylactic medical countermeasures exist to attenuate the symptoms that occur from radiation exposure. Manganese has recently been shown to be critical for radioresistance in a wide range of organisms. In this study, we designed a stringent feeding method to test the prophylactic effects of dietary manganese on *Drosophila*’s lifespan before exposure to acute irradiation. We found that male flies have substantially lower radioresistance than females, but feeding with low doses of MnCl_2_ before acute irradiation exposure extends male survival to that of females. Whole animal electron paramagnetic resonance analyses showed males have lower amounts of high-symmetry manganese-metabolite antioxidant complexes (H-Mn) than females, but manganese supplementation increases H-Mn to female levels. Levels of mitochondrial free-radical scavenger manganese-superoxide-dismutase 2 (MnSOD2) did not increase after acute irradiation, nor did loss of MnSOD2 sensitize larvae to acute irradiation exposure. These data support that prophylactic manganese feeding is sufficient to increase survivorship in males subjected to acute irradiation, independent of MnSOD2 levels, indicating a role of antioxidant manganese-metabolite H-Mn complexes for radioprotection. Furthermore, this *Drosophila* feeding method could be used to identify additional radiation countermeasures.

## 1. Introduction

Damaging exposure to acute ionizing radiation (IR) reduces cells’ capacities to clear damaged proteins, lipids, and other cellular components [1]. IR directly causes DNA base damage and double-strand breaks (DSB), and IR-induced oxidative damage to double-strand break repair proteins and compounds intensifies the effects of DNA damage [2]. Thus, mitotic cells in the bone marrow, gut, and reproductive organs are highly susceptible to IR, and it is these tissues that are the most sensitive in humans, particularly to acute doses [3]. IR exposure can occur from external sources, such as nuclear power plant disasters, and internally, such as from radon inhalation or contaminated diet. Acute radiation syndrome (ARS) results from exposures as low as 1 Gy, with the hematopoietic system, the gastrointestinal tract, and the cerebrovascular system, in particular, becoming damaged with increasing amounts of IR. FDA-approved therapeutic countermeasures currently exist for only hematopoietic damage: colony-stimulating factor and full bone marrow transplant [4]. There are currently no other identified therapeutics available for acute IR exposure.

**Figure 1:**
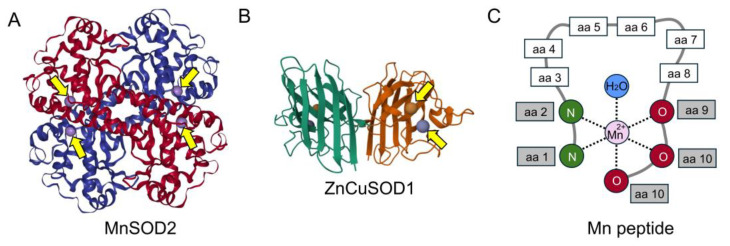
Enzymes and peptides critical for free radical clearance. (**A**) Crystal structure of human manganese-superoxide-dismutase 2 (SOD2) (PDB entry 1VAR). The purple spheres represent manganese ions (yellow arrows). (**B**) Crystal structure of canine Zn/CuSOD1 (PDB entry 7WWT). Orange spheres represent copper ions, and blue spheres represent zinc ions (arrows). Structures are from the worldwide Protein Databank [13]. (**C**) Example of a manganese (Mn) small peptide.

IR induces the formation of reactive oxygen species (ROS) through the splitting of water molecules by the high energy particles and the reduction of O_2_ to superoxide (O_2_^−^). Cells have natural defenses to ROS in the form of free radical scavenging enzymes. Superoxide-dismutase (SOD) family members catalyze the conversion of harmful superoxide radicals into hydrogen peroxide and oxygen [5]. Metals such as copper, zinc, and manganese are critical cofactors for the redox chemistry of SOD enzymes. In eukaryotes, MnSOD2 is a homotetramer mitochondrial protein that has four associated manganese ions (Figure 1A). Zn/CuSOD1 is a dimer located in the cytoplasm and has associated copper and zinc ions, which serve catalytic and structural roles, respectively (Figure 1B). In addition to enzymatic radical clearance, radioprotection is provided by non-enzymatic Mn^2+^-metabolite antioxidant complexes [6,7,8,9]. Recent studies have shed light on the potential of non-enzymatic high-symmetry, small-molecule complexes of manganese (H-Mn) to serve as effective, and possibly superior, catalysts for dismutating superoxide radicals into hydrogen peroxide and water (Figure 1C) [10,11,12]. Indeed, highly radioresistant organisms are correspondingly enriched in H-Mn complexes that form spontaneously with a variety of metabolites [10]. Thus, non-destructive electron paramagnetic resonance spectroscopy (EPR) and electron-nuclear double resonance (ENDOR) have accurately assessed the nature and abundance of these newly identified manganous antioxidants in organisms with a wide range of radioresistance and revealed that enhanced radioresistance of microbes correlates with increasing concentrations of H-Mn manganese-antioxidants [10]. Indirect evidence supports that manganous complexes can combat the effects of IR; SOD knockout strains of *Deinococcus radiodurans* and *C. elegans* have high concentrations of manganous complexes and exhibit remarkable radiation resistance, even surpassing that of wild type [11].

In an effort to identify radiomitigants, researchers have shown dietary administration of various agents is effective for radioprotection in mice and *Drosophila*. Mice fed melanin-containing mushrooms had greatly improved survival post-9 Gy IR as well as improved GI metrics 45 days post-irradiation [14]. Survival improvement post-IR has also been demonstrated with oral administration of antioxidants [15,16]. For example, N-acetyl-L-cysteine as a dietary supplement protected the intestinal epithelial barrier in mice after IR [17]. Mice ingesting *Aloe vera* extract had delayed radiation sickness symptoms and lower acid phosphatase and alkaline phosphatase levels in the liver after 6 Gy irradiation compared to control [18]. A mixture of ingested traditional Indian medicinal plants increased survival and improved DNA damage compared to control after 7.5 Gy exposure [19]. Feeding *Drosophila* with curcumin increased lifespan and decreased protein carbonylation following 20 Gy exposures [20]. Feeding flies with tea polyphenol or beta-carotene decreased mutation frequency and increased antioxidant levels post-10 Gy exposure compared to controls [21]. Finally, flies fed ibuprofen or two flavonoids (quercetin and epicatechin) showed increased lifespan compared to control after 1000 Gy exposure [22]. These studies support the idea that dietary supplements can ameliorate some effects of IR, but there is a need to develop a high-throughput model system to test the restorative properties of novel dietary sources with full lifespan analysis.

Here, we developed a *Drosophila* model to test dietary radioprophylaxis. We find that exposing young females and males to 700 or 1000 Gy gamma radiation had a sex-specific effect on lifespan, with males more sensitive than females. To test the dietary prophylactic potential of transition metal ions critical for antioxidant function, we designed a liquid feeder to facilitate ad libitum consumption of controlled liquid media. We find that prophylactic dietary treatment with MnCl_2_ extends lifespan post-IR in males but not in females, whereas CuCl_2_ and NiCl_2_ were ineffective. We also demonstrate, for the first time, EPR and ENDOR measurements on whole adult flies. This analysis revealed that the mechanism of action for Mn^2+^ treatment appears to be due primarily to non-enzymatic Mn^2+^-metabolite antioxidant complexes rather than levels of enzymatic MnSOD2. This was supported by our observation that *Drosophila* SOD2 protein levels remained constant with or without Mn^2+^ treatment and that loss of SOD2 did not sensitize larvae to the effect of IR. Overall, this study demonstrates a powerful tool for investigating dietary radioprotectants and provides evidence that manganese supplementation enhances male radioresistance through non-enzymatic antioxidant mechanisms. These findings underscore the potential of dietary interventions targeting Mn^2+^-based antioxidant pathways as a promising avenue for developing radioprotective strategies, particularly in contexts where enzymatic defenses are insufficient or compromised.

## 2. Materials and Methods

### 2.1. Irradiation and Dosimetry

Flies were exposed to gamma radiation in standard polystyrene *Drosophila* vials (Cat # 32-109, Genesee Scientific, El Cajon, CA, USA) capped with cellulose-acetate stoppers (Cat # 49-102, Genesee Scientific, El Cajon, CA, USA) containing standard cornmeal fly media. Six vials, two sets of matching triplicates, were irradiated per radiation treatment using a vendor-calibrated, ^137^Cs-sourced gamma irradiator (Shepard and Associates 68A Mark1, San Fernando, CA, USA). Dose rate = approximately 12.35 Gy/minute. Exposure length: 700 Gy = 57 min. 1000 Gy exposure = 81 min. Vials were placed on a rotating platform to ensure uniform exposure to the source emission. Consistent and precise dosimetry was maintained by calculating the length of every radiation exposure and accounting for daily source decay.

### 2.2. Feeding Protocols and Fly Strains

Fly strains used in this study: *w^1118^* and *Sod2^Δ02^*/*CyO Act GFP* (Bloomington Drosophila Stock Center, # 27643). For feeding experiments, freshly eclosed *w^1118^* adult female and male flies were collected and sex-separated, and 20 flies were distributed to liquid feeding chambers to administer supplemented and non-supplemented liquid medium. Consumption was characterized by a prominent “bluebelly” phenotype from the darkly stained medium. Control medium: 10% Sucrose (Cat # 84097, Millipore Sigma, St. Louis, MO, USA), 2.5% Bacto Yeast Extract (Cat # 212750, Thermo Fisher Scientific, Waltham, MA, USA), 0.5% FD&C Blue # 1 (Cat # IS15060, Aldon, Avon, NY, USA), and Supplemented media: Control medium with 5 µM or 10 µM manganese chloride (Cat # 221279, Millipore Sigma, St. Louis, MO, USA), 5 µM copper chloride (Cat # IS12161, Aldon, Avon, NY, USA), or 5 µM nickel chloride (Cat # 223387, Millipore Sigma, St. Louis, MO, USA). Feeding chambers were assembled from the bottom dish of a 60 mm petri plate covered with a 100 mL plastic beaker perforated with numerous holes to allow free exchange of air. Liquid medium dispensers were fashioned from 2 mL Eppendorf tubes by drilling 4 1/16 inch holes beneath the lip of the tube aperture. 1 mL of medium was dispensed to each tube, and a small piece of sponge was secured in the mouth of the tube. Thus, when inverted, the liquid in the feeding tube permeates the sponge, flowing to the drilled openings without spilling out of the tube. Flies were allowed to feed ad libitum for 2 days. After 2 days, flies were transferred to conventional *Drosophila* vials filled with cornmeal medium [8.25% corn syrup (Karo Light Corn Syrup, ACH Food Companies, Oakbrook Terrace, IL, USA), 5% corn meal (Cat # 62-100, Genesee Scientific, El Cajon, CA, USA), 1.25% dry inactivated yeast (Cat # 62-103, Genesee Scientific, El Cajon, CA, USA), 0.75% soy flour (Cat # 62-115, Genesee Scientific, El Cajon, CA, USA), 0.5% propionic acid (Cat # P1386, Millipore Sigma, St. Louis, MO, USA), 0.1% Tegosept (Cat # 20-258, Genesee Scientific, El Cajon, CA, USA)] and promptly irradiated with 700 or 1000 Gy gamma radiation. Flies were monitored daily for lifespan and transferred to fresh vials every 2–3 days. Experiments were performed in triplicate with 20 flies in each replicate. All flies were housed at room temperature with a 12-h light cycle.

### 2.3. Lifespan Analysis

Twenty male and female *w^1118^* adults were monitored daily after irradiation for their full lifespan, which was performed minimally in triplicate. Since irradiation weakens animals, flies were considered dead when they no longer reacted to a repeated physical stimulus. To perform statistical analysis, daily survivorship data were compiled and entered into the Online Application for Survival Analysis 2 (OASIS, [23]) (https://sbi.postech.ac.kr/oasis2/) (accessed on 10 October 2024). Statistical significance between control and experiment was determined using the Wilcoxon–Breslow–Gehan test.

### 2.4. Pupation Assay

Three replicates of twenty-first instar larvae (*w^1118^*, *Sod2^Δ2^*/*CyO Act GFP* (*Sod2^Δ2^*/+), and *Sod2^Δ2^*) were carefully placed in standard fly food vials at room temperature (day 1). Control vials for all three genotypes were not subject to irradiation. Rates of pupation and eclosion were determined by counting the number of pupae or adult flies daily, respectively. Experimental vials were irradiated with 100 Gy on day 5 when the larvae had reached the third instar but were still feeding and had not crawled up the sides of the vial. Pupation and eclosion data were analyzed statistically using GraphPad Prism (Dotmatics, Boston, MA, USA), and the data were analyzed statistically with ANOVA with multiple comparisons followed by Tukey’s post hoc test. Photographs of pupae were taken using an Accu-scope 3076 digital microscope 0.67×–4.5× (Accu-Scope, Commack, NY, USA).

### 2.5. Western Blot Analysis

Western blotting was performed as previously described [24,25]. Twenty males and females were exposed to 700 Gy, allowed to recover for 24 h, and then frozen. Protein samples from adult flies were extracted in a 1X SDS sample buffer (50 mM Tris-Cl, pH 6.8, 2% SDS, 0.1% Bromophenol blue). Extract from the same number of flies was loaded on 4–15% TGX gels (Cat # 4561086, Bio-rad Laboratories, Hercules, CA, USA) and transferred to a nitrocellulose membrane (Cat # 88018, Thermo Fisher Scientific, Waltham, MA, USA) using a Trans-blot Semi-dry apparatus (Cat # 1073940, Bio-rad Laboratories, Hercules, CA, USA). The membrane was incubated in Ponceau stain (Cat # P7170, Millipore Sigma, St. Louis, MO, USA) for 3–5 min, then imaged. After blocking in 5% nonfat milk, blots were probed against appropriate primary and horseradish peroxidase (HRP) conjugated secondary antibodies. The blots were then exposed to SuperSignal West Pico PLUS Chemiluminescent Substrate (Cat # 34580, Thermo Fisher Scientific, Waltham, MA, USA and detected on X-films developed using an automated X-ray film processor (PROTEC GmbH & Co. KG, Oberstenfeld, Germany). Images from developed X-rays were captured using a color scanner, saved as JPGs, and converted to grayscale images using Photoshop (version 26.2). No other manipulation occurred. We used anti-SOD2 (1:1000, Cat # LS-B3694-50, LSBio Shirley, MA, USA) for the western blot. Quantification was performed in triplicate using ImageJ (version 1.54j)/FIJI (version 2.9.0) open-sourced software [26] with SOD2 bands normalized against Ponceau labeling. The standard deviation was calculated using GraphPad-PRISM (version 10.1.2) software. *p* values were calculated in GraphPad-PRISM software using an unpaired *t*-test.

### 2.6. Electron Paramagnetic Resonance Analysis of Flies

Quartz EPR tubes (ID 2 mm, OD 2.5 mm, ATS Life Sciences, Vineland, NJ, USA) were filled with 70% ethanol from the base to the top by threading a length of 1 mm diameter silicone tubing attached to the 22 G needle of a 1 mL syringe into the base of the tube and withdrawing it as the tube filled. 2-day old treated and untreated flies were collected and anaesthetized with CO_2_ gas. The wings of the flies were carefully removed, and the flies were agitated briefly in 70% ethanol to wet them and dislodge any air bubbles trapped in their fine body hairs. The flies were then deposited into the mouth of the tube one at a time until sinking to the bottom. Ten to twelve flies were loaded into the EPR tube one by one to a height (12–15 mm) that filled the active volume (10 mm) of the EPR cavity, thus maximizing the response and assuring a uniform reading among samples. The tube was then flushed several times with 20% glycerol with similarly inserted silicone tubing and syringe. The loaded tubes were then placed immediately on dry ice to vitrify samples in solution and stored at −80 °C. EPR experiments performed on duplicate EPR tubes provided reproducible results within experimental error (see Figure S3).

35-GHz (Q-band) continuous wave absorption-display field swept EPR spectra were collected on a modified Varian Associates E109 spectrometer equipped with a Varian E110 microwave bridge (Varian, Inc., Paolo Alto, CA, USA) as described previously [27]. Data acquisition was performed using a lab-written program in LabVIEW (National Instruments Corp, Austin, TX, USA) with the following experimental conditions: MW frequency 34.9 GHz, temperature 2 K, modulation amplitude 2 G, modulation frequency 100 kHz, time constant 64 ms, scan rate 1 kG/min).

EPR spectra simulations were performed using EasySpin EPR spectrum simulation package in MATLAB (version R2024b) (MathWorks, Natick, MA, USA) [28]. As detailed in [9], the Mn^2+^ EPR spectrum of fly samples could be partitioned into the percentage of a narrow component associated with antioxidant metabolite complexes of Mn^2+^, designated H-Mn, and that of a broad component associated with Mn-enzymes including MnSOD2, denoted, L-Mn. The EPR spectrum of the H-Mn EPR pool displays six sharp peaks at the center (due to ^55^Mn electron-nuclear hyperfine splitting) with narrow wings that are reproduced with a small zero-field splitting parameter, D~1000 MHz/0.03 cm^−1^. The broad L-Mn component is simulated with D~4000 MHz/0.12 cm^−1^. The ratio of integrated intensities of the absorption-display EPR spectra (area under the curve) of two samples corresponds to their relative concentrations of Mn^2+^. With L-Mn as a minor (and essentially unvarying) contribution to the EPR intensity for all fly samples, then the relative changes in Mn^2+^ upon supplementation and the relative Mn^2+^ populations of males and females are simply calculated by ratioing the H-Mn amplitudes (total area multiplied by H-Mn percentage), (see Figures S1 and S2).

Pulsed ENDOR experiments were performed on a lab-built Q-band 35 GHz pulse spectrometer that employs SpecMan4EPR software (version 3.4 CS 64bit) (specman4epr.com) for data collection [29,30]. ^31^P ENDOR spectra were collected using Refocussed Mims (reMims) four-pulse sequence [22] with the following typical experimental conditions: Magnetic Field 1.2194 T; MW frequency, 34.769 GHz; reMims pulse sequence, π/2 − τ1 − π/2 − T − π/2 − τ2 − π − (τ1 + τ2)-echo, where (π/2) = 30 ns; τ = 120 ns; RF pulse, T = 20 us; τ1 = 200 ns; τ2 = 400 ns; Repetition Time = 10 ms; T = 2 K. The ENDOR spectra are normalized to their Mn^2+^ EPR intensity, and thus, ^31^P ENDOR peak amplitudes are a direct measure of bound Pi.

## 3. Results

### 3.1. Prophylactic Dietary MnCl_2_ Extends Lifespan in Males Exposed to IR

**Figure 2:**
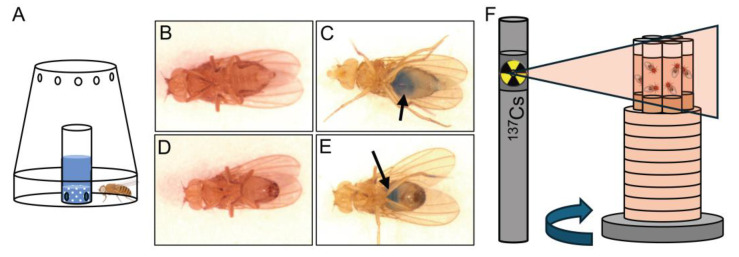
Method for prophylactically feeding radiomitigators to *Drosophila.* (**A**) Schematic of the feeding apparatus. (**B**–**E**) Dorsal view of representative females (**B**,**C**) and males (**D**,**E**). After one day of feeding, the blue dye is seen in the guts ((**B**,**D**) vs. (**C**,**E**), arrows). (**F**) Schematic of irradiation setup. Six fly vials (three experimental groups and three control groups) containing standard food rotate on a platform.

**Figure 3:**
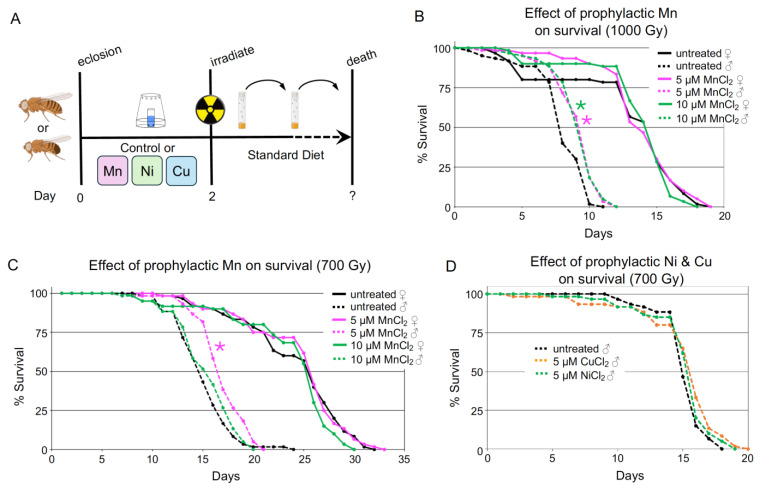
Prophylactic feeding of MnCl_2_ protects males from acute radiation exposure. (**A**) Schematic of prophylactic feeding regimen. (**B**,**C**) Graphs of *w^1118^* males (♂) and females (♀) fed zero, 5, or 10 µM MnCl_2_ then irradiated with 1000 Gy (**B**) or 700 Gy (**C**). Treatment with MnCl_2_ extended the lifespan of males (dashed lines) but not females (solid lines). (**D**) Graph of males fed 5 µM CuCl_2_ or NiCl_2_ then exposed to 700 Gy. Neither treatment increased lifespan compared to untreated. Graphs were plotted using Microsoft Excel. Each point on the lifespan represents the average of triplicates of twenty flies that were irradiated simultaneously. Statistical analysis was calculated using Online Application for Survival Analysis 2 (OASIS), and statistical significance was calculated using the Wilcoxon–Breslow–Gehan test. * = *p* < 0.02 compared to untreated.

Flies readily consumed experimental liquid diets, as confirmed by observation of blue-stained intestines in both females and males. (Figure 2A–E, arrows). Placement of six vials of fly samples surrounding a central empty vial on a rotating platform at a dosimetrically calibrated position from a ^137^Cs source for timed exposures determined by daily half-life decay calculation produced reliable and consistent lifespan data for all groups and treatments (Figure 2F). We first examined the effect of gamma radiation on the average lifespan of untreated male and female files to determine control values. We found that females exposed to 1000 Gy lived for approximately 18 days, whereas females exposed to 700 Gy lived for 30 days, about twice as long (Figure 3B,C, solid lines). We found males were much more sensitive to IR exposure than females. Males exposed to 1000 Gy lived for approximately 10 days, whereas males exposed to 700 Gy survived for around 20 days (Figure 3B,C, dashed lines).

Next, we supplemented the liquid diet with MnCl_2_ at two different concentrations (5 µM and 10 μM) for two days pre-irradiation. Manganese-treated females with either concentration show no change in their lifespan post-radiation for both 1000 Gy and 700 Gy exposures (Figure 3B,C, magenta and green solid lines). In contrast, manganese-treated males exhibited significantly improved radiation survival. After 1000 Gy exposure, both 5µM and 10 µM concentrations of MnCl_2_ statistically lengthened male lifespans (Figure 3B, magenta and green dashed lines). After 700 Gy exposure, 5 μM MnCl_2_ statistically lengthened lifespans, whereas 10 μM did not show improvement (Figure 3C, magenta dashed line vs. green dashed line). This is supported by both the mean and median lifespan increasing with MnCl_2_ treatment (Table 1). Additionally, we tested prophylactic feeding of copper and nickel for survivorship increase in males, as both transition metals are cofactors for Zn/CuSOD1 and NiSOD, respectively. However, treatment with neither metal conferred lifespan extension in males exposed to 700 Gy (Figure 3D). These data support that a brief period of dietary MnCl_2_ before irradiation is sufficient to significantly extend the lifespan of male fruit flies.

### 3.2. Total Manganese and Manganese Speciation Correlates with Radioresistance in Drosophila

**Figure 4:**
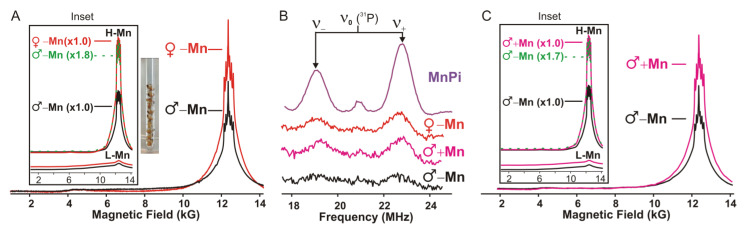
EPR and ENDOR characterization of manganese speciation in *Drosophila.* (**A**) 35 Gigahertz absorption-display EPR spectra comparing male (black, ♂−Mn) and female (red, ♀−Mn) adult flies, along with a photo of flies in the EPR tube. Inset: Decomposition of the Mn^2+^ EPR spectrum of male and female flies into two contributions, H-Mn and L-Mn (also see Figure S1). From duplicate experiments, females contain almost twice (1.8x) the relative levels of H-Mn compared to males. For details on decompositions, see Materials and Methods and Figure S1. (**B**) 35 GHz Refocused Mims (reMims) pulsed ^31^P ENDOR, doublets centered at the Larmor frequency and split by the hyperfine coupling frequency (A ≅ 4 MHz); samples are indicated on the right. See Materials and Methods for conditions. (**C**) 35 Gigahertz absorption-display EPR spectra comparing untreated (♂−Mn, black line) and MnCl_2_ treated males (♂+Mn, magenta line). Inset: Partitioning of EPR spectra into H-Mn and L-Mn, with treated males having relative ~1.7x more H-Mn than untreated males, with similar overall Mn^2+^ amounts from duplicate experiments. For experimental conditions, see Materials and Methods.

In view of the above findings that female fruit flies demonstrate greater radiation survivorship than the males, we performed electron paramagnetic resonance (EPR) and electron-nuclear double resonance (ENDOR) on *w^1118^* non-IR adult males and females to measure the amount and speciation of Mn^2+^ antioxidants. Mn^2+^ EPR spectra for both *w^1118^* male (♂−Mn) and female (♀−Mn) flies can be decomposed into two contributions. The first contribution is from a pool of low-molecular-weight antioxidant complexes of Mn^2+^ with orthophosphate, histidine, and carboxylate ligands (H-Mn), which display narrow EPR spectra with characteristic six sharp peaks at the center (Figure 4A, Inset, H-Mn, and Figure S1). These species are known to scavenge and detoxify superoxide [6,7,8]. The second contribution is from the L-Mn pool, which primarily contains Mn-enzymes, including MnSOD2, and whose spectra have broad wings (Figure 4A, Inset, L-Mn). To complement this assessment of H-Mn, ^31^P ENDOR measurements give the percentage of the H-Mn that is present as the orthophosphate (Pi) complex of Mn^2+^ (Mn-Pi), a well-known excellent antioxidant [31]. For these measurements, whole adults were placed in glycerol inside quartz EPR tubes.

The Q-band 2K absorption-display EPR spectra show that adult females (♀−Mn), which are less sensitive to IR, have almost twice the amount of Mn^2+^ compared to males (♂−Mn). Simulations that partition the EPR spectra of females and males into the contributions from H-Mn and L-Mn (Figure 4A, Inset, Figure S1) show that both male and female flies contain mostly H-Mn, with only a small and similar total population of enzymatic Mn complexes (L-Mn) (Figure 4A, red vs. black lines). Ratioing the H-Mn amplitudes shows that the females contain almost twice the amount of H-Mn (1.8x) as the males (Figure 4A, Inset, see Materials and Methods for details). In addition, ^31^P Refocused Mims ENDOR analysis showed that the fraction of H-Mn present as Mn-Pi in the females is twice that of the males (Figure 4B, red vs. black). Thus, the EPR and ENDOR together show that the amount of Mn-Pi in females (♀−Mn) is roughly four-fold higher than that in males (♂−Mn) (Figure 4B). In short, the EPR/ENDOR-derived whole-body average Mn-speciation reveals that females on a normal diet have a higher concentration of H-Mn antioxidants (~double), with a higher percentage of H-Mn population present as the Mn-Pi complex (~four-fold), while MnSOD levels in males and females are similar (Figure 4A). The higher concentration of the antioxidant H-Mn species helps explain why female flies (♀−Mn) display higher radiation resistance than males (♂−Mn) (Figure 3B,C).

As males showed enhanced lifespan with prophylactic feeding of MnCl_2_, but females did not (Figure 3), we compared the Mn^2+^ content and speciation of untreated (♂−Mn) vs. treated (♂+Mn) males. Partitioning of the EPR spectra (Figure 4C, Inset) shows that Mn^2+^ treatment of male flies led to an increase in H-Mn concentration (~1.7x), such that it approaches the population of the untreated females (♀−Mn), with no increase in MnSOD2 L-Mn (Figure 4C, magenta vs. black lines, Figures S1 and S2), while ENDOR shows that the percentage of the Pi complex is likewise increased (Figure 4B). Overall, these observations support the conclusion that females (♀−Mn) are more radioresistant compared to males (♂−Mn) in part because they have more and likely more effective antioxidant H-Mn, while prophylactically feeding males (♂+Mn) MnCl_2_ enhances their radiation survival by increasing both the quantity and efficacy of H-Mn in the animals.

### 3.3. Dietary MnCl_2_ Increases Male Lifespan Independent of Superoxide-Dismutase 2 Levels

**Figure 5:**
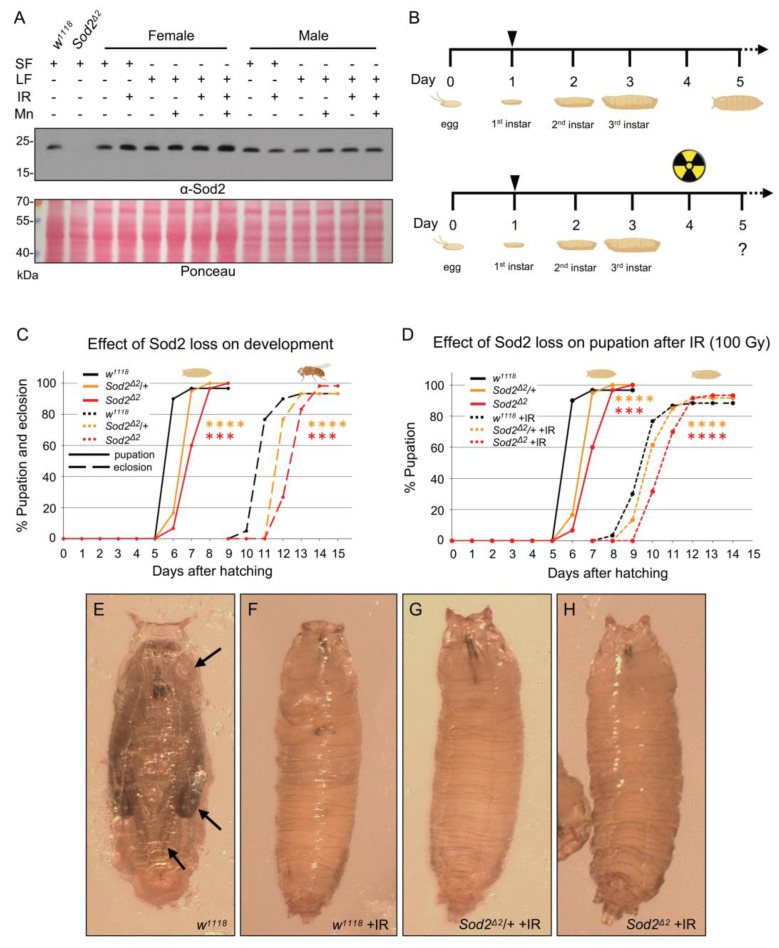
MnCl_2_ prophylaxis is not due to increased SOD2 (**A**) Western blot analysis of SOD2 levels in adult flies. SF—standard food, LF—liquid food, IR = 700 Gy irradiation, Mn = LF + 10 µM MnCl_2_. The null mutant *Sod2^Δ2^* is a control for SOD2 antibody specificity. Ponceau is shown as a loading control. Blots were performed in triplicate. (**B**) Experimental design for assessing the developmental effects of loss of *Sod2* without (**top**) and with (**bottom**) exposure to acute irradiation. The arrow indicates when larvae were collected. (**C**) Graph showing results of pupation and eclosion assay for unirradiated *w^1118^*, *Sod2^Δ2^*/+, and *Sod2^Δ2^* mutants. (**D**) Graph showing results of pupation assay for *w^1118^*, *Sod2^Δ2^*/+, and *Sod2^Δ2^* mutants after exposure to 100 Gy during feeding third instar larval stage. The data for non-irradiated larvae (solid lines) are the same data from panel (**C**). (**E**–**H**) Pupae from larvae irradiated with 100 Gy during feeding third instar larval stage neither developed nor eclosed. (**E**) *w^1118^* pupa with normally developed eyes, wings, and legs (arrows). *w^1118^* (**F**), *Sod2^Δ2^*/+ (**G**), and *Sod2^Δ2^* (**H**) pupae from irradiated larvae did not develop. Data in (**C**,**D**) were graphed and analyzed using Microsoft Excel. Significance was calculated using two-way ANOVA followed by Tukey’s post hoc test. *** = *p* < 0.001, **** = *p* < 0.0001. *p* values are compared with *w^1118^*.

MnSOD2 is a mitochondrially-localized manganese-dependent superoxide-dismutase capable of scavenging superoxide radicals (Figure 1A) [9]. We wanted to determine whether the improved lifespan we observed with dietary MnCl_2_ treatment was due to altered *Drosophila* MnSOD2 (also called SOD2) levels. For this, we used western blot analyses to probe for SOD2 levels in females and males (Figure 5A). We used *Sod2* null mutants (*Sod2^Δ2^*) and found that SOD2 was absent as expected. We then manipulated the flies in various ways by changing the food source (standard food (SF) vs. liquid food (LF)), whether they were irradiated (IR), and whether they were treated with MnCl_2_ (Mn). Examining females and males fed on a liquid diet, we found that neither IR nor dietary MnCl_2_ caused a change in SOD2 levels (Figure 5A and Figure S4). These observations support that the lifespan protective effect of dietary MnCl_2_ is not due to increased concentrations of MnSOD2, consistent with the EPR observation that the L-Mn population is not increased.

### 3.4. Loss of MnSOD2 Increases Sensitivity of Larvae to the Effects of IR

The presence of MnSOD2 is reported to be unnecessary in conferring a protective effect against IR in *C. elegans* or *Deinococcus radiodurans* [5]. A quintuple knockout for MnSOD in *C. elegans* and a knockout of SODA in *D. radiodurans* were not more sensitive to IR and, in fact, had better radiation survival compared to the wild type. We wanted to determine whether this was also the case for *Drosophila Sod2* mutants. As *Sod2^Δ2^* mutant adults live only 24 h after eclosing, we examined pupation and eclosion rates for larvae. We first examined developmental rates for *w^1118^*, *Sod2^Δ2^*/+, and *Sod2^Δ2^* mutants without IR (Figure 5B, top, C). Homozygous *Sod2^Δ2^* mutants had delayed pupation and eclosion compared to *w^1118^* (Figure 5C, red vs. black lines), with heterozygotes pupating and eclosing at an intermediate rate (Figure 5C, orange vs. black lines). To determine whether SOD2 was dispensable for acute radiation sensitivity, we collected first instar larvae of all three genotypes and irradiated them with 100 Gy at the feeding third-instar stage (Figure 5B, bottom). IR delayed pupation for all three genotypes compared to non-irradiated larvae in a manner similar to non-irradiated larvae (Figure 5D, dashed lines vs. solid lines). *Sod2^Δ2^* mutants had the greatest delay compared to *w^1118^* (Figure 5D, red vs. black dashed lines), and again, heterozygous *Sod2^Δ2^*/+ larvae had an intermediate delay compared to *w^1118^* (Figure 5D, orange vs. black dashed lines). With exposure to 100 Gy IR, no genotype exhibited signs of pupal development, nor did any eclose (Figure 5E vs. Figure 5F–H). Thus, SOD2 presence did not add additional protection for larvae from the effects of acute IR.

## 4. Discussion

### 4.1. Developing Drosophila as a Robust and Stringent Model to Identify Radioprophylactics Against the Effects of Acute Irradiation

Eukaryotes have well-conserved cellular pathways; thus, studies in model systems, including *Drosophila*, have contributed important knowledge to how cells function. Gamma irradiation fundamentally damages all cells similarly due to the properties of radiation chemistry [32,33]. *Drosophila* have historically been an excellent model to study the effects of radiation [20,34,35,36,37,38]. The advantages of using flies to test the effectiveness of radiation countermeasures include the high number of individuals that can be tested and the plethora of available genetic and cell biological tools. In addition, since *Drosophila* live on average a maximum of one hundred days, the effect of IR on survival can be monitored for their entire lifespan. We chose to investigate dietary prophylaxis because the gastrointestinal tract is susceptible to multiple aspects of IR injury. First, the stem cell population, which maintains the intestinal epithelium, is highly susceptible to IR due to its rapid rate of division [39]. Second, species within the influential microbiota of the intestinal microbiome are radiosensitive [40]. Third, the lumen of the intestine is an aqueous mixture of biomaterials that is also susceptible to the formation of ROS. Thus, we speculate that dietary radioprophylactic agents are protective against IR due to their ability to deliver an intervention to all three vulnerable components of the gastrointestinal tract.

### 4.2. Dietary Manganese as a Prophylactic Treatment for IR Exposure in Drosophila

Highly radiation-resistant organisms have high levels of Mn^2+^, with increasing radioresistance correlating with increased levels [10,11]. In eukaryotes, the Mn^2+^ conversion of superoxide into hydrogen peroxide occurs within the MnSOD2 protein. However, Mn^2+^ can bind metabolites and small peptides to form antioxidant complexes that also effectively clear free radicals [6,7,8,41,42]. Small manganous complexes carry out the same reactions as MnSOD2 while allowing Mn^2+^ to be more easily accessible to accept electrons from free radicals, thus potentially making them equally or even more effective than MnSOD2. Mn-Pi antioxidant complexes dismutate superoxide anions through direct donation and acceptance of electrons to and from superoxide anions [43]. Pi facilitates the accumulation of Mn^2+^ ions cytosolically, preventing reactions with other biomolecules. Thus, Mn-Pi antioxidants are capable of attenuating superoxide anions generated throughout the cell.

We compared Mn^2+^ content and speciation between male and female *Drosophila* using whole animal EPR/ENDOR. Using intact animals ensures that Mn species are not degraded or altered during sample processing. With this method, females were found to have approximately twice the amount of Mn^2+^ in the form of H-Mn species compared to males, and Mn-treated males also accumulated higher levels of H-Mn compared to untreated males. In addition, SOD2 levels did not increase after IR or MnCl_2_ treatment. These results suggest that the mechanism of action for prophylaxis is an increase in Mn antioxidant complexes. While Mn^2+^ treatment was effective in males, it is important to note that high levels of Mn can be neurotoxic. Mn toxicity appears to be related to the point of entry, with the gut able to regulate absorption [44,45]. Millimolar concentrations of dietary heavy metals, including Mn^2+^, for five to fifteen days were surprisingly tolerated [46]. In mice, initial experiments have shown preliminary data that injected MnCl_2_ confers prophylactic protection to IR with no obvious neurological effects, supporting the potential efficacy of dietary treatment in mammals [47].

### 4.3. Future Directions and Unanswered Questions

There are currently no FDA-approved radioprophylactics, despite their critical importance in protecting emergency responders, warfighters, astronauts, and the general public from the harmful effects of IR. To address this gap, we have developed a protocol that capitalizes on the advantages of the *Drosophila* model to facilitate rapid and effective testing of compounds with potential radioprotective influence. Using this system, we have identified MnCl_2_ as a promising candidate. Our data support the hypothesis that the oral administration of Mn^2+^ drives a radioprotective increase in antioxidant manganous-metabolite complexes, a mechanism that warrants further investigation. Future studies will focus on identifying specific organs protected by Mn^2+^, with particular emphasis on the gut, given *Drosophila*’s established utility as a model for gastrointestinal pathology. Additionally, our screening method will be leveraged to characterize a broader range of compounds for their IR-protective properties.

## Figures and Tables

**Table 1:** Detailed lifespan analysis comparing irradiated and treated *Drosophila*.

Subject/Dose/Treatment	Minimum ^a^	Maximum ^a^	Mean ^a^	Median ^a^
Female-700 Gy-0 µM MnCl_2_	14	32	22.45	26
Female-700 Gy-5 µM MnCl_2_	14	33	22.85	26
Female-700 Gy-10 µM MnCl_2_	11	30	21.95	26
Male-700 Gy-0 µM MnCl_2_	11	24	13.2	15
Male-700 Gy-5 µM MnCl_2_	13	21	15.7	17
Male-700 Gy-10 µM MnCl_2_	9	20	14.1	16
Female-1000 Gy-0 µM MnCl_2_	5	20	11.6	16
Female-1000 Gy-5 µM MnCl_2_	9	20	13.8	15.5
Female-1000 Gy-10 µM MnCl_2_	6	19	12.1	16
Male-1000 Gy-0 µM MnCl_2_	3	12	7.95	9
Male-1000 Gy-5 µM MnCl_2_	6	13	9.15	11
Male-1000 Gy-10 µM MnCl_2_	6	13	9.15	11

^a^ Days.

## Data Availability

All data are contained within the manuscript or Supplementary Figures. The original contributions presented in this study are included in the article/Supplementary Material. Further inquiries can be directed to the corresponding author.

## Supplementary Materials

The following supporting information can be downloaded at https://www.mdpi.com/article/10.3390/antiox14020134/s1. Figure S1: 35 GHz EPR spectra of male and female flies without Mn supplementation, partitioned into H-Mn and L-Mn contributions, as described in [10] and the main text; see Figure 4A, Inset. EPR contributions (%): H-Mn ~75%, L-Mn ~25% for males, ~85% H-Mn, ~15% L-Mn for females, with overall Mn^2+^ in female flies 1.8x that of males on standard food. For conditions, see Materials and Methods. Figure S2: 35 GHz EPR spectra of male flies with and without MnCl_2_ supplementation, partitioned into H-Mn and L-Mn contributions, as described in [10] and the main text; see Figure 4C. EPR contributions (%): H-Mn ~80%, L-Mn ~20% for males, ~88% H-Mn, ~12% L-Mn for Mn supplemented males, with the overall amount in supplemented flies 1.7x that of males on the unsupplemented diet. For conditions, see Materials and Methods. Figure S3: 35 GHz absorption-display CW EPR spectra comparing (A) *w^1118^* males (triplicates), (B) *w^1118^* males with diets supplemented with 5 μM MnCl_2_ (triplicates), and (C) *w^1118^* females (duplicates). Sample 1—*w^1118^* males (A, black trace)—and Sample 2—Mn-supplemented males (B, red trace)—showed EPR intensity very different from their partner duplicates due to the mispositioning of flies in the EPR tube. Thus, further analysis employed averages of the two equivalent spectra for each condition. Both samples of *w^1118^* females had a good alignment, and their average was used. Conditions: Microwave frequency, 34.973 GHz; modulation amplitude, 2 G; time constant, 64 ms; T = 2 K. Figure S4: Neither irradiation (IR) nor MnCl_2_ treatment with liquid diet affected Sod2 levels. The quantification of Sod2 levels normalized to Ponceau staining was graphed using GraphPad-PRISM software. LF—liquid food, IR = 700 Gy irradiation, Mn = 10 μM MnCl_2_. Error bars: Standard deviation (S.D.) calculated in GraphPad-PRISM software. Statistical significance was calculated in GraphPad-PRISM software using an unpaired *t*-test.
