# Supplementary material for: Prophylactically Feeding Manganese to Drosophila Confers Sex-Specific Protection from Acute Ionizing Radiation Independent of MnSOD2 Levels
Source: Antioxidants (Basel). 2025 Jan 23;14(2):134. doi: 10.3390/antiox14020134 (PMC11851552; doi:10.3390/antiox14020134)
Supplement: Supplementary file 1 [file antioxidants-14-00134-s001.zip › antioxidants-3390288-supplementary.pdf]

## Supplementary Material

## Supplemental Figure S1

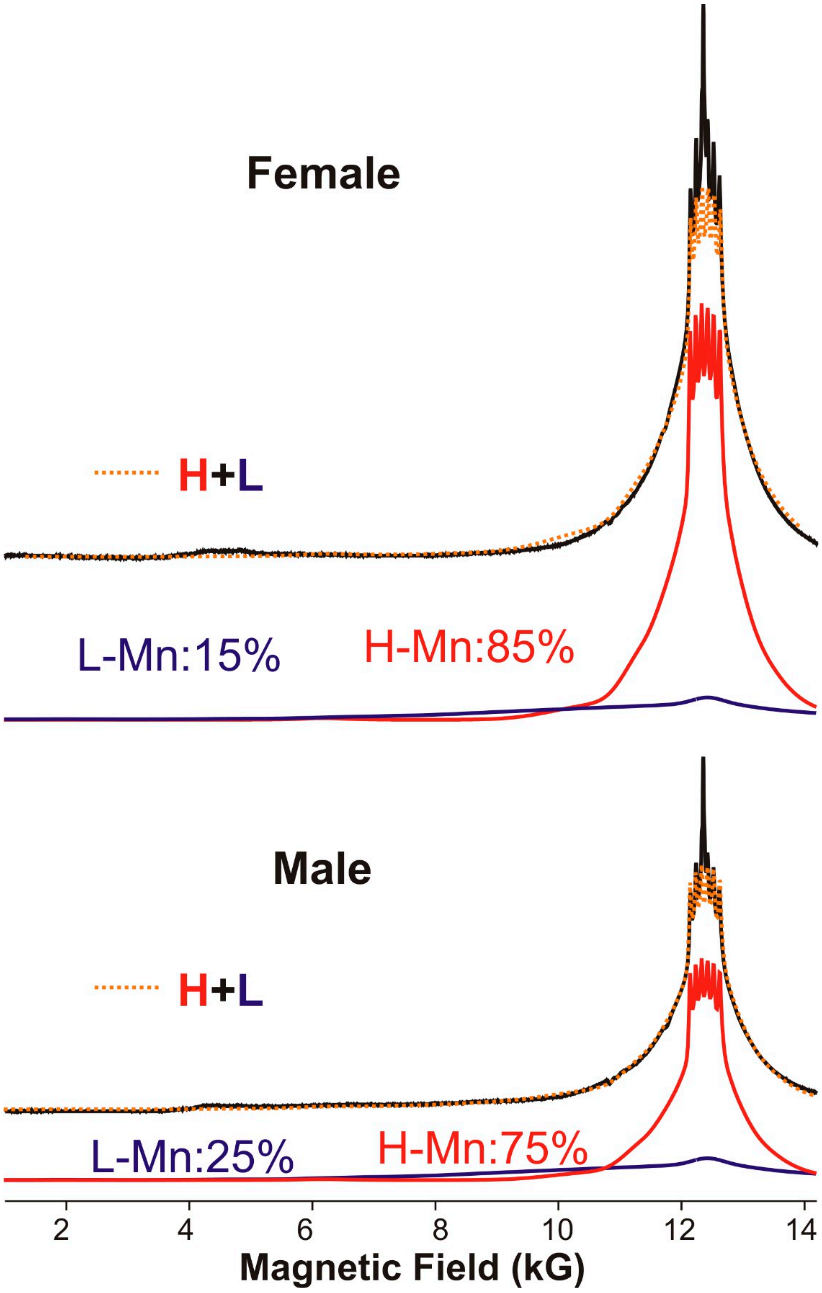


**Figure S1:** 35 GHz EPR spectra of male and female flies without Mn supplementation, partitioned into H-Mn and L-Mn contributions, as described in [11] and the main text; see Fig 4A, Inset. EPR contributions (%): H-Mn ~75%, L-Mn ~25% for males, ~85% H-Mn, ~15% L-Mn for females, with overall $Mn^{2+}$ in female flies 1.8x that of males on standard food. For conditions, see materials and methods.

## Supplemental Figure S2

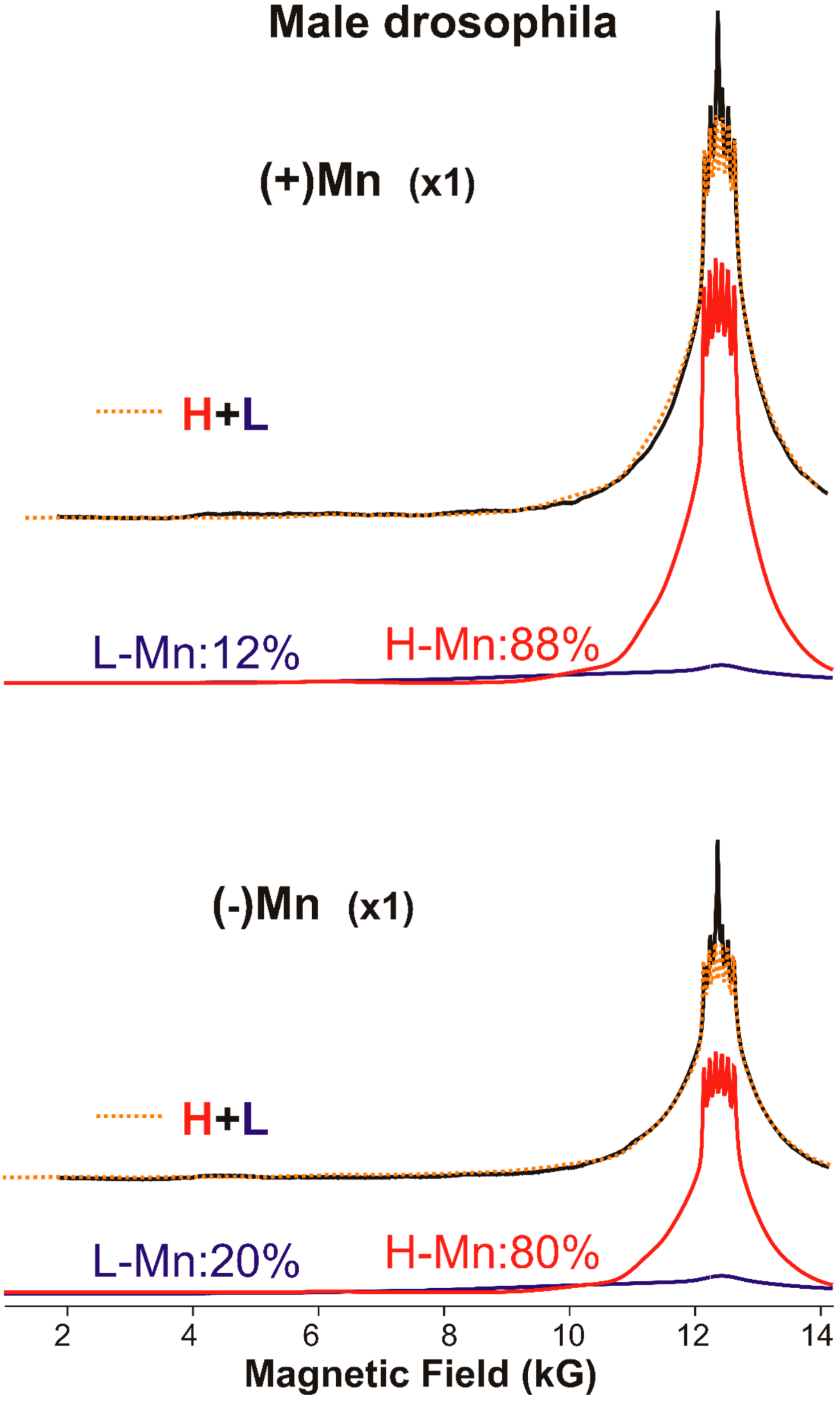


**Figure S2:** 35 GHz EPR spectra of male flies with and without $MnCl_2$ supplementation, partitioned into H-Mn and L-Mn contributions, as described in [11] and the main text; see Fig 4C. EPR contributions (%): H-Mn ~80%, L-Mn ~20% for males, ~88% H-Mn, ~12% L-Mn for Mn supplemented males, with overall amount in supplemented flies 1.7x that of males on unsupplemented diet. For conditions, see materials and methods.

## Supplemental Figure S3

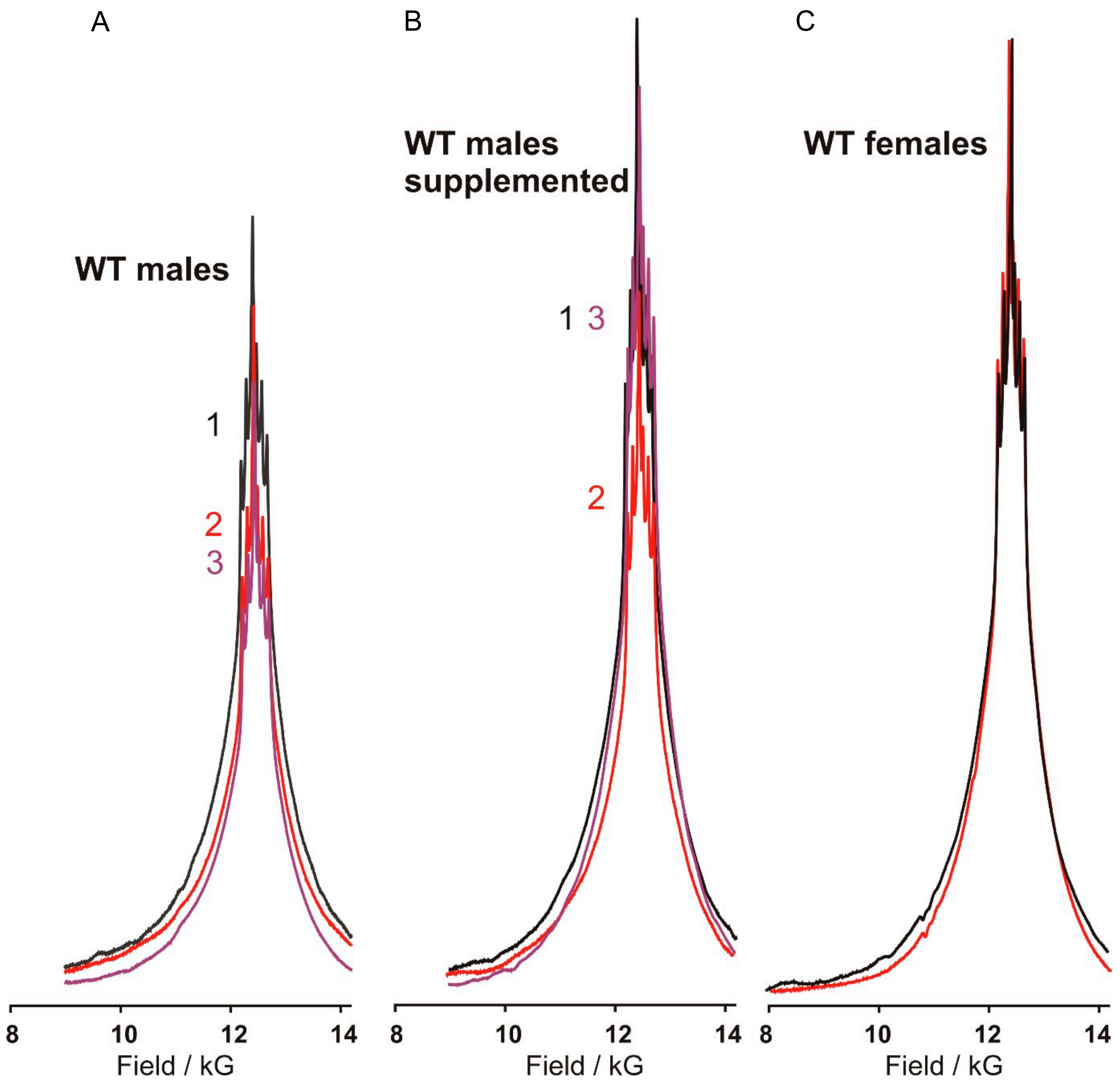


**Figure S3:** 35 GHz absorption-display CW EPR spectra comparing (A) $w^{1118}$ males (triplicates), (B) $w^{1118}$ males, with diets supplemented with 5 µM $MnCl_2$ (triplicates), and (C) $w^{1118}$ females (duplicates). Sample 1 of $w^{1118}$ males (A, black trace) and sample 2 of Mn-supplemented males (B, red trace) showed EPR intensity very different from their partner duplicates due to mispositioning of flies in the EPR tube. Thus, further analysis employed averages of the two equivalent spectra for each condition. Both samples of $w^{1118}$ females had good alignment, and their average was used. *Conditions*: Microwave frequency, 34.973 GHz; modulation amplitude, 2 G; time constant, 64 ms; T = 2 K.

## Supplemental Figure S4

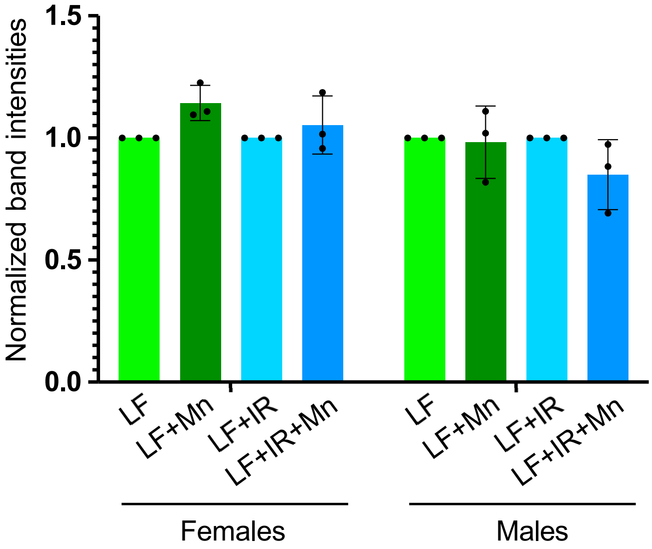


**Figure S4:** Neither irradiation (IR) nor $MnCl_2$ treatment with liquid diet affected Sod2 levels. Quantification of Sod2 levels normalized to Ponceau staining graphed using GraphPad-PRISM software. LF - liquid food, IR = 700 Gy irradiation, Mn = 10 µM $MnCl_2$. Error bars: Standard deviation (S.D.) calculated in GraphPad-PRISM software. Statistical significance was calculated in GraphPad-PRISM software using an unpaired t-test.
